# Adverse Outcome in COVID-19 Is Associated With an Aggravating Hypo-Responsive Platelet Phenotype

**DOI:** 10.3389/fcvm.2021.795624

**Published:** 2021-12-10

**Authors:** Waltraud C. Schrottmaier, Anita Pirabe, David Pereyra, Stefan Heber, Hubert Hackl, Anna Schmuckenschlager, Laura Brunnthaler, Jonas Santol, Kerstin Kammerer, Justin Oosterlee, Erich Pawelka, Sonja M. Treiber, Abdullah O. Khan, Matthew Pugh, Marianna T. Traugott, Christian Schörgenhofer, Tamara Seitz, Mario Karolyi, Bernd Jilma, Julie Rayes, Alexander Zoufaly, Alice Assinger

**Affiliations:** ^1^Department of Vascular Biology and Thrombosis Research, Centre of Physiology and Pharmacology, Medical University of Vienna, Vienna, Austria; ^2^Department of General Surgery, Division of Visceral Surgery, Medical University of Vienna, General Hospital Vienna, Vienna, Austria; ^3^Institute of Physiology, Centre of Physiology and Pharmacology, Medical University of Vienna, Vienna, Austria; ^4^Institute of Bioinformatics, Biocenter, Medical University of Innsbruck, Innsbruck, Austria; ^5^Department of Medicine IV, Clinic Favoriten, Vienna, Austria; ^6^Institute of Cardiovascular Sciences, College of Medical and Dental Sciences, University of Birmingham, Birmingham, United Kingdom; ^7^Institute of Immunology and Immunotherapy, University of Birmingham, Birmingham, United Kingdom; ^8^Department of Clinical Pharmacology, Medical University of Vienna, General Hospital Vienna, Vienna, Austria

**Keywords:** COVID-19, platelet, infection, platelet dysfunction, platelet-leukocyte interaction, outcome, platelet activation and reactivity

## Abstract

Thromboembolic complications are frequently observed in Coronavirus disease 2019 (COVID-19). While COVID-19 is linked to platelet dysregulation, the association between disease outcome and platelet function is less clear. We prospectively monitored platelet activation and reactivity in 97 patients during the first week of hospitalization and determined plasma markers of platelet degranulation and inflammation. Adverse outcome in COVID-19 was associated with increased basal platelet activation and diminished platelet responses, which aggravated over time. Especially GPIIb/IIIa responses were abrogated, pointing toward impeded platelet aggregation. Moreover, platelet-leukocyte aggregate formation was diminished, pointing toward abrogated platelet-mediated immune responses in COVID-19. No general increase in plasma levels of platelet-derived granule components could be detected, arguing against platelet exhaustion. However, studies on platelets from healthy donors showed that plasma components in COVID-19 patients with unfavorable outcome were at least partly responsible for diminished platelet responses.

Taken together this study shows that unfavorable outcome in COVID-19 is associated with a hypo-responsive platelet phenotype that aggravates with disease progression and may impact platelet-mediated immunoregulation.

## Introduction

COVID-19 is a viral disease characterized by acute respiratory distress and systemic involvement of different pathophysiologies (1, 2), with impaired hemostasis being a major determinant of outcome. Thromboembolic events are frequently (27–46%) reported in patients suffering from COVID-19, with a particularly high incidence in patients requiring ICU treatment (3–7). Thromboembolic complications are reported both at venous and arterial sites (4), rendering the underlying coagulopathy a complex phenomenon arising from the interface between platelet malfunction and derangement of the coagulation cascade (8). Patients with COVID-19-associated coagulopathy (CAC) display slightly reduced platelet counts accompanied by increased levels of D-dimer and a prolongation in prothrombin time (9).

The precise role of platelets in CAC and the underlying mechanism of reduced platelet counts are still incompletely understood but low platelet count is associated with disease severity (10–13) and platelet apoptosis was observed in COVID-19 patients requiring ICU treatment (14).

Compared to healthy controls or patients with other pulmonary infections patients with severe COVID-19 display elevated markers of platelet activation including increased thromboxane A_2_ (TXA_2_) release (12, 15), surface expressed CD62P and CD63 (12, 15–18), activated glycoprotein (GP) IIb/IIIa (12, 19, 20), decreased intraplatelet serotonin (5-HT) and platelet factor 4 (PF4/CXCL4) (21) with increased plasma levels of 5-HT, PF4, and soluble CD40 ligand (sCD40L) (19, 21). Of note, not all studies detected differences in platelet activation in dependency of disease severity (21) or ICU requirement (17).

Currently, the link between platelet function and COVID-19 associated mortality is still unclear. While one study found plasma levels of CD62P, CD40L and TXA_2_ to be independently associated with all-cause mortality (22), two other studies could not detect differences in platelet activation between non-fatal and fatal disease (15, 21).

Since COVID-19 patients represent a very heterogeneous population with different co-morbidities, longitudinal studies of platelet functions might be better suited to understand platelet malfunctions in these patients. Therefore, we monitored COVID-19 patients with different disease severities, ranging from asymptomatic to critically diseased, during their hospital stay. We found that fatal outcome was associated with elevated basal platelet activation and impaired platelet reactivity, in particular regarding GPIIb/IIIa activation. This profound hypo-reactivity was at least partially mediated by plasma components as platelets from healthy donors became hypo-reactive in the presence of COVID-19 patient plasma.

## Materials and Methods

Please also see Supplementary Methods in the Online Supplement.

### Ethics Approval

The study was approved by the local Ethics Committee (Medical University of Vienna: EK1315/2020 and EK1548/2020) and carried out in accordance with the Declaration of Helsinki. Participants gave written informed consent.

### Prospective Study Cohort

This study was carried out as part of the Austrian Coronavirus Adaptive Clinical Trial (ACOVACT; ClinicalTrials.gov NCT04351724).

All patients with confirmed SARS-CoV-2 infection admitted to the Clinic Favoriten, the primary COVID-19 hospital in Vienna, Austria, between 17th April 2020 and 28th October 2020 were asked to participate in this study if study inclusion was possible within 72 h after hospital admission. Inclusion criteria were SARS-CoV-2 infection confirmed by real-time PCR of naso- or oropharyngeal swab and ≥18 years of age. Exclusion criteria were life expectancy under 1 month (e.g. due to severe comorbidities), pregnancy or breast feeding, stage 4 kidney disease and severe liver dysfunction which could affect hemostasis. No virus variants were found within the study cohort.

Blood was drawn upon inclusion (day 0) and every 2-3 days over 1 week for flow cytometric analysis of platelet function or multiplex/ELISA analysis of plasma components. Additional blood draws for prospective analyses which were not part of clinical routine were omitted if a patient was anemic (hemoglobin <11 g/dl).

### Patient Sample Procurement

Blood was drawn from the antecubital vein using 21G needles into vacutainer tubes containing citrate or citrate, theophylline, adenosine and dipyridamole (CTAD) as specified samples not used for clinical routine. Venipuncture was always done during morning rounds by the same personnel who covered both general and ICU ward in order to prevent differences in blood collection between patients with different clinical presentation and/or outcome. The samples were immediately transported at room temperature to the biosafety level 2 facility at the Institute of Vascular Biology and Thrombosis Research, Medical University of Vienna (<1 h), where samples were also immediately analyzed.

### Flow Cytometry

Citrate-anticoagulated whole blood was stimulated with different concentrations (0 μM, 0.6 μM, 3 μM and 6 μM) of adenosine diphosphate (ADP) or thrombin-receptor activating peptide 6 (TRAP-6) for 15 min, stained with primary labeled antibodies for 20 min and diluted with 1-step Fix/Lyse solution (eBioscience). Samples were measured on a Cytoflex S cytometer within 6 h and analyzed using CytExpert 2.4 software (both Beckman Coulter). Antibody cocktails are detailed in Supplementary Table 1.

### Plasma Preparation

Plasma of citrate and CTAD anticoagulated blood was prepared as previously described to ensure minimal platelet pre-activation (23). Briefly, blood was stepwise centrifuged, first for 10 min at 1.000 g at 4 °C, followed by high speed centrifugation (10 min, 10.000 g) of the supernatant to clear remaining platelets and debris. Plasma was aliquoted and stored at−80 °C until analysis without further freeze/thaw cycles.

### *In vitro* Platelet Activation in Patient Plasma

Citrate-anticoagulated blood from naïve healthy donors not previously exposed to SARS-CoV-2 (confirmed by IgG serology) was centrifuged for 20 min at 120 g to obtain platelet-rich plasma (PRP). Platelets were subsequently pelleted for 90 s at 1.000 g in the presence of prostacyclin (PGI_2_, 0.1 μg/ml) and resuspended in PBS at double density (500 μl PBS per ml PRP). Concentrated platelets were diluted 1:8 with patient plasma before stimulation with cross-linked collagen-related peptide (CRP-XL; 50 ng/ml, 15 min; CambCol Laboratories). Platelets were stained with α-CD62P-BrilliantViolet605 (1:100) and PAC1-FITC (1:60) for 20 min before fixation in 1% formaldehyde and flow cytometric analysis. Plasma samples were obtained from matched patients with different outcome that did not receive anti-platelet medication.

### ELISA and Multiplex Analysis

Multiplex analysis was done using pre-defined LegendPlex bead-based immunoassay panels thrombosis, fibrinolysis, vascular inflammation 2, and proinflammatory chemokines (all BioLegend). Assays were performed according to manufacturer's instructions, measured on a Cytoflex S cytometer (Beckman Coulter) and analyzed using LegendPlex v8.0 software (BioLegend). Plasma activity of ADAMTS13 and vWF were determined by ELISA using Technozym ADAMTS13 activity kit (Technoclone) and REAADS vWF activity test kit (Corgenix) according to manufacturer's instructions.

### Statistics and Data Presentation

Statistical evaluation and graphical presentations were performed with IBM SPSS 27 or GraphPad Prism 8. Metric data were tested for Gaussian distribution by Kolmogorov-Smirnov and Shapiro-Wilk test and differences between multiple groups analyzed by one-way ANOVA or Kruskal Wallis Test. Differences between metric data differing in two factors were analyzed by mixed-effects model with Geisser-Greenhouse correction. Nominal data were analyzed by Fisher's exact test and correlations of platelet activation markers by partial regression analysis. Violin plots show median (line) and quartiles (dotted line), timelines show median values with interquartile range.

## Results

### Characterization of the Patient Cohort

**Figure 1:**
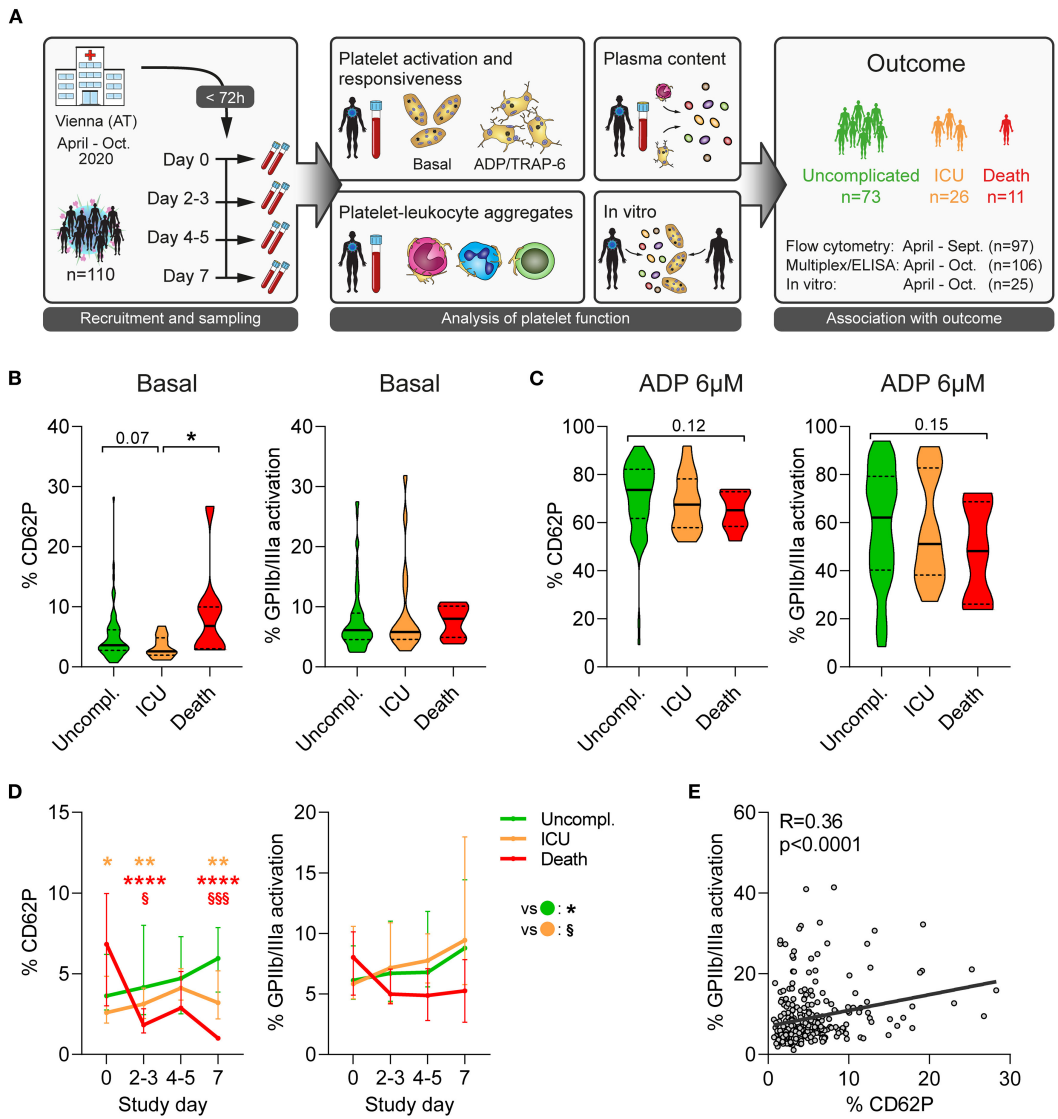
Unfavorable outcome in COVID-19 is associated with declining platelet activity. **(A)** Study design: 110 patients admitted to the primary COVID-19 hospital in Vienna, Austria, were included in this longitudinal study within 72 h after hospital admission and prospectively analyzed. Blood was collected every 2-3 days over 1 week to determine platelet function and elucidate outcome-specific differences. **(B,C)** Platelet activation upon study entry at **(B)** basal condition and **(C)** after stimulation with 6μM ADP (15 min) was assessed in 97 patients upon hospital admission by quantifying surface CD62P expression and GPIIb/IIIa activation (PAC1 antibody binding). **(D)** Basal platelet activation was monitored over the span of 1 week after study. Asterisks (^*^) indicate significant differences to uncomplicated (orange: ICU; red: death), section signs (^§^) indicated significant differences between ICU and death. **(E)** Correlation of basal CD62P expression and GPIIb/IIIa activation of platelets. *n* = 97 patients. ^*^p < 0.05, ^**^p < 0.01, ^****^p < 0.0001; ^§^*p* < 0.05, ^§§§^*p* < 0.001.

The effect of COVID-19 on platelet activation has been investigated in various studies, however the dynamic changes of platelet dysfunction over disease progression and their association with different disease outcome have not been addressed yet. Therefore, we prospectively analyzed 110 patients (≥18 years, hemoglobin >11 g/dL) with confirmed SARS-CoV-2 infection who were admitted at the primary COVID-19 hospital in Vienna, Austria (Clinic Favoriten) between April and October 2020 and evaluated their platelet function during the first week of hospitalization (Figure 1A). Of note, as national policy demanded that all hospitalized patients were tested for SARS-CoV-2 irrespective of medical complaints, our cohort also comprises 11 patients (10.0%) without symptoms at the time of admission.

Higher disease severity at admission was associated with worse outcome, however 5% of patients that were initially classified as mild or moderate also died in hospital (Table 1). Therefore, we stratified the cohort according to outcome: 73 patients (66.4%) showed uncomplicated course of disease, 26 (23.6%) required ICU treatment and 11 patients (10.0%) died (Table 1). Complicated disease course (ICU requirement, death) was associated with differences in age and presence of comorbidities of the pulmonary system such as chronic obstructive pulmonary disease and asthma (Table 1). Laboratory findings (D-dimer, prothrombin time, international normalized ratio and activated partial thromboplastin time) at admission failed to show a clear dysregulation of coagulation parameters in patients with complicated disease, which might be influenced by standard treatment with prophylactic anticoagulation. Notably, platelet counts were similar between outcome groups, though prevalence of anti-platelet therapy was slightly but not significantly higher in non-survivors than overall (36.4% vs 15.5%) (Table 2).

**Table 1:** Patient demographics.

	**Missing data**	**All (*n =* 110)**	**Uncomplicated (*n =* 73)**	**ICU (*n =* 26)**	**Death (*n =* 11)**	
**Parameter**	***n***	***n*** **(%) Median (IQR)**	***n*** **(%) Median (IQR)**	***n*** **(%) Median (IQR)**	***n*** **(%) Median (IQR)**	***p*** -value^*^
**Sex**	–					*p =* 0.301
Male		72 (65.5)	46 (63.0)	20 (76.9)	6 (54.5)	
Female		38 (34.5)	27 (37.0)	6 (23.1)	5 (45.5)	
Age (years)	–	62 (49–77)	61 (48–76)	59 (49–64)	80 (79–86)	***p*** **<** **0.001**
**Comorbidities**						
Current smoker	32	6 (7.7)	6 (11.5)	0 (0.0)	0 (0.0)	*p =* 0.327
Obesity (BMI > 25)	12	54 (55.1)	34 (54.0)	13 (52.0)	7 (70.0)	*p =* 0.707
Diabetes type II	–	25 (22.7)	17 (23.3)	5 (19.2)	3 (27.3)	*p =* 0.829
Hypertension	1	55 (50.5)	34 (46.6)	14 (53.8)	7 (70.0)	*p =* 0.352
Cardiovascular disease (any)	–	26 (23.6)	16 (21.9)	7 (26.9)	3 (27.3)	*p =* 0.732
Coronary heart disease	–	14 (12.7)	9 (12.3)	4 (15.4)	1 (9.1)	*p =* 0.903
Chronic heart failure	–	8 (7.3)	6 (8.2)	2 (7.7)	0 (0.0)	*p =* 1.000
Atrial fibrillation	–	11 (10.0)	8 (11.0)	2 (7.7)	1 (9.1)	*p =* 1.000
Peripheral arterial disease	–	6 (5.5)	4 (5.5)	1 (3.8)	1 (9.1)	*p =* 0.653
Chronic obstructive pulmonary disease	–	14 (12.7)	7 (9.6)	2 (7.7)	5 (45.5)	***p*** **<** **0.008**
Asthma	1	6 (5.5)	3 (4.2)	0 (0.0)	3 (27.3)	***p** **=*** **0.011**
Hypo- / Hyperthyroidism	1	12 (11.0)	8 (11.1)	3 (11.5)	1 (9.1)	*p =* 1.000
Chronic renal insufficiency	–	14 (12.7)	11 (15.1)	2 (7.7)	1 (9.1)	*p =* 0.741
Chronic liver disease	–	4 (3.6)	2 (2.7)	1 (3.8)	1 (9.1)	*p =* 0.383
Malignancy	–	12 (10.9)	6 (8.2)	3 (11.5)	3 (27.3)	*p =* 0.149
**Medication (anti-platelet/anticoagulation)**						
Anti-platelet therapy	–	17 (15.5)	9 (12.3)	4 (15.4)	4 (36.4)	*p =* 0.128
Anticoagulation therapy	–	108 (98.2)	71 (97.3)	26 (100.0)	11 (100.0)	*p =* 1.000
**COVID-19 classification at admission** ^†^	–					***p** **=*** **0.012**
Asymptomatic / mild		15 (13.6)	12 (16.4)	2 (7.7)	1 (9.1)	
Moderate		52 (47.3)	38 (52.1)	10 (38.5)	4 (36.4)	
Severe		31 (28.2)	20 (27.4)	6 (23.1)	5 (45.5)	
Critical		12 (10.9)	3 (4.1)	8 (30.8)	1 (9.1)	
**Clinical characteristics**						
Time from admission to discharge/death (days)	1	13 (9–23)	12 (9–19)	23 (17–33)	8 (6–14)	***p*** **<** **0.001**
Invasive ventilation	–	5 (4.5)	0 (0.0)	2 (7.7)	3 (27.3)	***p*** **<** **0.001**
**Prospective analysis**						
Cytokine profiling		106	69	26	11	***p** **=*** **0.050**
Flow cytometric analysis		97	70	19	8	*p =* 0.200
*In vitro* experiments		23	8	8	7	***p** **=*** **0.001**

* *p < 0.05. Nominal variables were compared using Fisher's exact test, metric variables were compared using Kruskal Wallis test*;

† *COVID-19 classification according to the guidelines issued by the World Health Organization in mild (fever <38°C, no dyspnea, no pneumonia), moderate (fever, respiratory symptoms, pneumonia), severe (respiratory distress with respiratory rate ≥30 per min, oxygen saturation <93% at rest) and critical (respiratory failure with requirement of mechanical ventilation, requirement of ICU); BMI, body mass index; ICU, intensive care unit; IQR, interquartile range. Bold values highlight statistically significant data*.

**Table 2:** Laboratory findings at admission.

	**Missing data**	**All (*n =* 110)**	**Uncomplicated (*n =* 73)**	**ICU (*n =* 26)**	**Death (*n =* 11)**	
**Parameter**	***n***	**Median (IQR)**	**Median (IQR)**	**Median (IQR)**	**Median (IQR)**	***p*** -value^*^
Hemoglobin (g/dL)	3	13.2 (12.2–14.5)	13.3 (12.2–14.7)	13.8 (12.6–14.7)	12.2 (11.2–13.0)	***p** **=*** **0.069**
Red blood cell count (x0^12^/L)	3	4.6 (4.1–5–0)	4.6 (4.1–5.1)	4.7 (4.4–5.1)	4.2 (3.5–4.3)	***p** **=*** **0.019**
Platelet count (x10^9^/L)	3	182 (146–235)	179 (146–239)	182 (137–224)	198 (161–245)	*p =* 0.777
Leukocyte count (x10^9^/L)	3	6.0 (4.2–7.5)	5.4 (3.8–6.8)	6.8 (5.4–9.0)	6.5 (5.2–8.8)	***p** **=*** **0.029**
Lymphocyte count (x10^9^/L)	10	0.8 (0.6–1.2)	0.9 (0.6–1.3)	0.8 (0.7–1.1)	0.7 (0.5–0.8)	*p =* 0.157
Neutrophil count (x10^9^/L)	10	4.5 (3.1–6.0)	3.9 (2.9–5.5)	5.2 (3.6–8.0)	5.0 (3.8–7.1)	***p** **=*** **0.038**
Monocyte count (x10^9^/L)	10	0.3 (0.2–0.5)	0.3 (0.2–0.5)	0.3 (0.2–0.4)	0.4 (0.3–0.6)	*p =* 0.138
Eosinophil count (x10^9^/L)	10	0.01 (0.00–0.03)	0.01 (0.00–0.04)	0.01 (0.00–0.01)	0.01 (0.00–0.03)	*p =* 0.443
Basophil count (x10^9^/L)	10	0.02 (0.01–0.04)	0.02 (0.01–0.04)	0.02 (0.02–0.03)	0.02 (0.01–0.03)	*p =* 0.427
C-reactive protein (mg/L)	3	59.5 (32.8–88.8)	55.0 (25.7–77.4)	83.8 (59.5–112.6)	57.4 (34.1–140.0)	***p** **=*** **0.017**
D-dimer (mg/L)	21	0.7 (0.5–1.1)	0.7 (0.5–1.1)	0.6 (0.4–1.0)	1.4 (0.6–3.6)	*p =* 0.265
Prothrombin time (%)	7	100.0 (89.7–109.6)	99.9 (88.6–111.4)	101.4 (91.4–110.4)	96.9 (91.6–103.1)	*p =* 0.653
International normalized ratio	8	1.0 (1.0–1.1)	1.0 (1.0–1.1)	1.0 (1.0–1.1)	1.0 (1.0–1.1)	*p =* 0.752
Activated partial thromboplastin time (s)	14	32.6 (29.1–36.9)	32.7 (29.1–37.2)	32.4 (28.8–33.8)	31.0 (30.2–37.9)	*p =* 0.799

* *p < 0.05. Metric variables were compared using Kruskal Wallis test; ICU, intensive care unit; IQR, interquartile range. Bold values highlight statistically significant data*.

### Patients With Fatal Outcome Exhibit Elevated Markers of Platelet Activation Upon Study Enrollment

We first investigated platelet activation at the time of study entry. While a higher disease severity score at admission was not associated with elevated platelet activation (Supplementary Figure 1), CD62P expression was significantly higher in fatal outcome than upon ICU requirement (Figure 1B). Further, patients with fatal outcome showed a trend toward increased CD40L and CD63 expression and GPIIb/IIIa activation compared to those with non-fatal outcome, indicating augmented platelet degranulation and fibrinogen binding capacity (Figure 1B and Supplementary Figure 2A).

To determine platelet reactivity, we stimulated patient blood with different concentrations of adenosine diphosphate (ADP) or thrombin receptor activator peptide 6 (TRAP-6) and assessed platelet activation. Contrary to the elevated basal activation levels, platelets of patients with fatal disease were less responsive to agonist stimulation, in particular regarding GPIIb/IIIa activation. Though data did not reach statistical significance (Figure 1C and Supplementary Figure 2B, data not shown), they suggest that fatal COVID-19 outcome is associated with hyper-active but hypo-reactive platelets.

As reduced platelet density and induction of coagulation could impact platelet function, we examined kinetics of platelet count and coagulation parameters in patients with different outcome after hospitalization, but we could detect no outcome-specific differences in platelet count, prothrombin time, international normalized ratio nor activated partial thromboplastin time (Supplementary Figure 3).

### Platelet Hypo-Responsiveness in Patients With Fatal Outcome Worsens Over Time

Although platelets of patients with fatal disease outcome exhibited increased basal activation upon study entry, markers of degranulation and integrin activation declined rapidly (Figure 1D and Supplementary Figure 4A). Indeed, already 2-3 days after study entry CD62P expression was significantly lower in fatal disease than non-fatal disease (Figure 1D).

In order to mitigate variation caused by different time points of study entry after admission or by missing values, we assessed kinetics of platelet activation and agonist response in a mixed model approach. While degranulation markers CD40L and CD63 correlated moderately well with CD62P (*R* = 0.72 and *R* = 0.60, respectively), GPIIb/IIIa activation did not (*R* = 0.36) (Figure 1E and Supplementary Figure 4B) and we thus used CD62P and GPIIb/IIIa for modeling.

**Figure 2:**
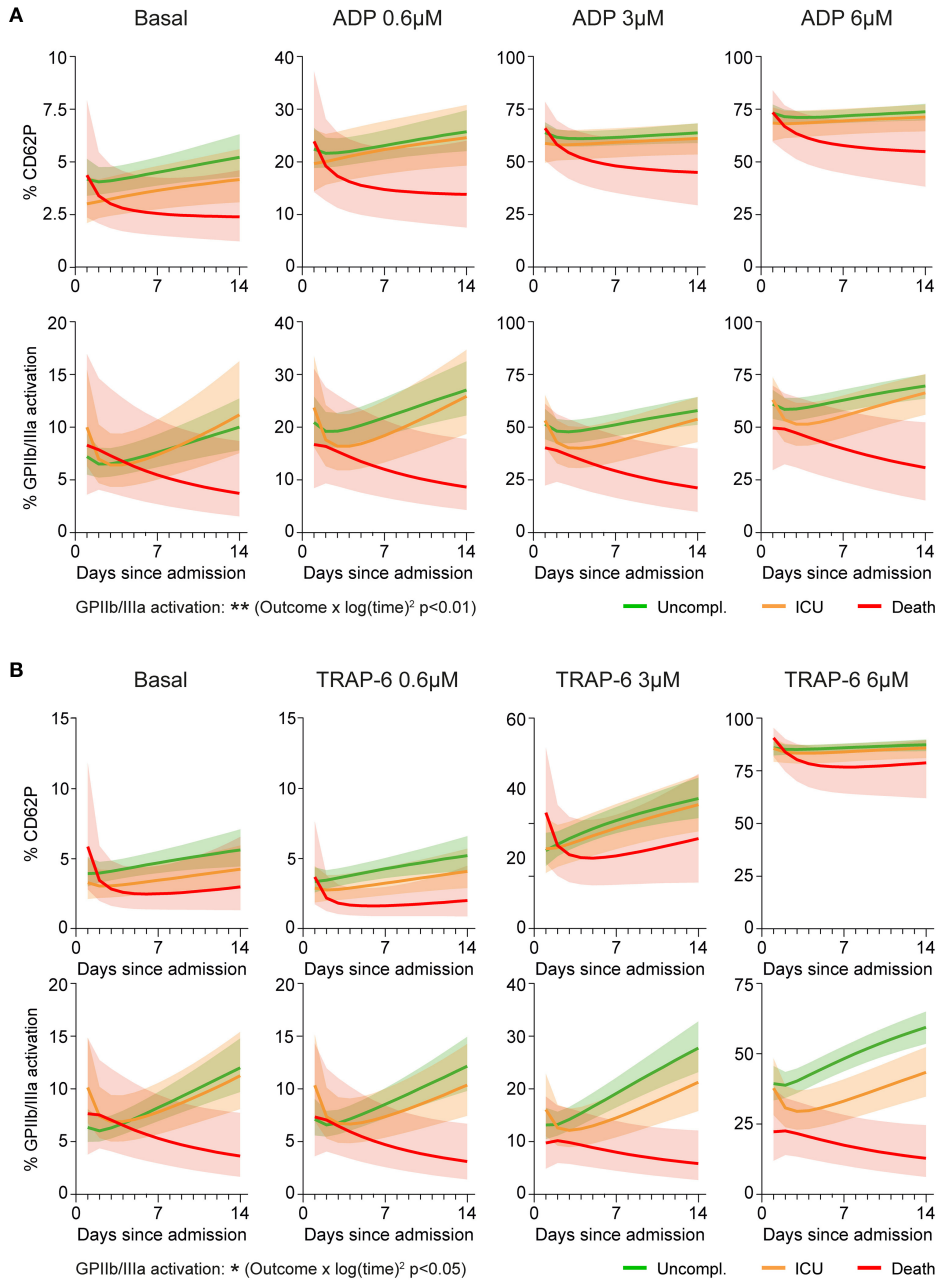
Aggravating platelet hypo-responsiveness in fatal COVID-19 abrogates GPIIb/IIIa activation. Platelet activation at basal condition and in response to agonist stimulation (0.6–6μM; 15 min) was monitored in 97 patients during hospitalization by determining surface CD62P expression and GPIIb/IIIa activation (PAC1 antibody binding). A mixed model approach was used to estimate the different kinetics of platelet activation and reactivity over time between patients with different outcomes. The model was applied independently to explore **(A)** ADP-triggered CD62P expression (upper panel), ADP-triggered GPIIb/IIIa activation (lower panel), **(B)** TRAP-6-triggered CD62P expression (upper panel) and TRAP-6-triggered GPIIb/IIIa activation (lower panel). Lines indicate central tendencies of patient groups with their 95% confidence intervals (shaded areas). *n* = 97 patients. ^*^p < 0.05, ^**^p < 0.01.

CD62P responses to different ADP concentrations showed equal kinetics in the outcome groups (all interactions with time *p* > 0.45), whereas ADP-triggered GPIIb/IIIa activation significantly differed between outcome groups (interaction ‘outcome^*^log(time)^2^’ *p* < 0.01) (Figure 2A and Supplementary Figure 5). Overall, patients with non-fatal disease showed increasing ADP-induced GPIIb/IIIa activation over time, whereas platelet activation and reactivity in fatally-ill patients gradually declined during hospitalization.

Similarly, TRAP-6-induced CD62P responses were not significantly different between outcomes (all interactions with time *p* > 0.06), whereas kinetics of GPIIb/IIIa activation varied significantly (interaction ‘outcome^*^log(time)^2^’ *p* < 0.05) (Figure 2B and Supplementary Figure 6), with fatally-ill patients showing progressive platelet hypo-reactivity.

Of note, fibrinogen levels were slightly increased in ICU patients relative to uncomplicated disease throughout the observation period (Supplementary Figures 7A,B). However, fatal outcome was not associated with altered fibrinogen levels and we could not observe a correlation between plasma fibrinogen and platelet GPIIb/IIIa activation (Supplementary Figure 7C).

### Impaired GPIIb/IIIa Activation in Patients With Fatal COVID-19 Is Partly Mediated by Plasma Factors

**Figure 3:**
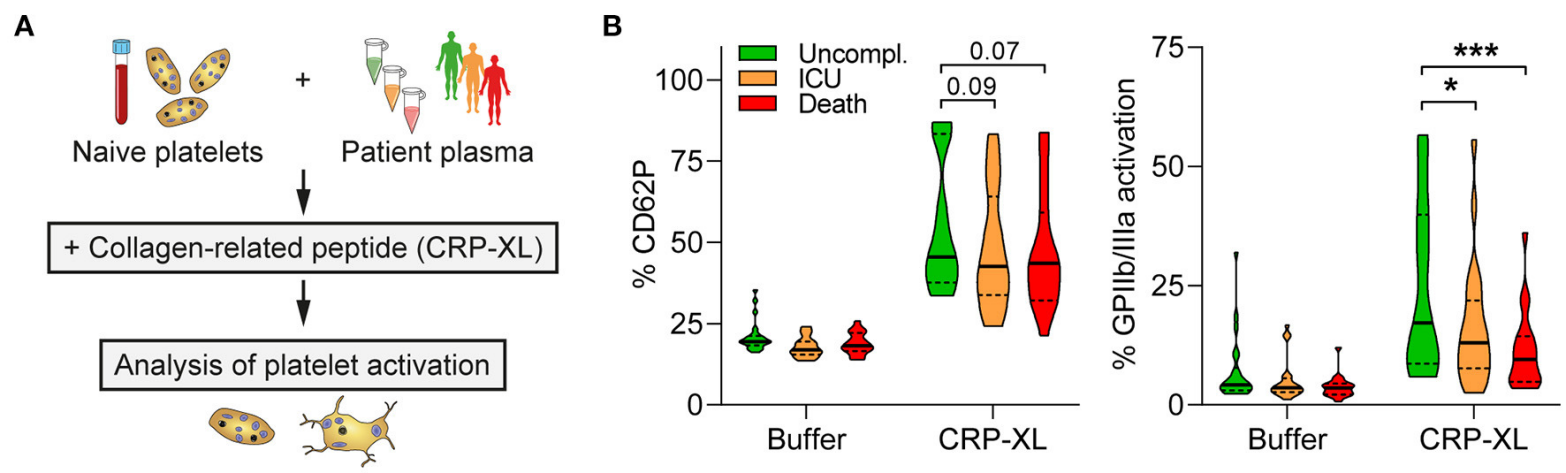
Platelet hypo-reactivity is partially plasma-mediated. **(A)** Experimental setup: Naïve platelets of healthy donors were incubated with plasma of COVID-19 patients with different outcome (10 min), followed by CRP-XL stimulation (50 ng/ml, 15 min) and evaluation of platelet activation. **(B)** Surface CD62P expression and GPIIb/IIIa activation (PAC1 antibody binding) determined *via* flow cytometry. *n* = 3 platelet donors/7-8 patient plasma per outcome. ^*^p < 0.05, ^***^p < 0.001.

Next, we elucidated if diminished GPIIb/IIIa activation in fatal COVID-19 was due to changes in platelet production or mediated by the microenvironment. We isolated platelets from naïve healthy donors, incubated them with plasma from COVID-19 patients (day 0, no anti-platelet medication) and analyzed platelet activation in response to cross-linked collagen-related peptide (CRP-XL) (Figure 3A). Unfavorable COVID-19 outcome (ICU, death) of the plasma donor was associated with reduced platelet responsiveness compared to uncomplicated disease. While CD62P expression was not quite significantly affected, CRP-XL-induced GPIIb/IIIa activation was significantly higher in plasma from patients with uncomplicated disease than in plasma from patients that required ICU treatment or died (Figure 3B). Thus, our data demonstrate that platelet hypo-reactivity in fatally-ill COVID-19 patients was at least partially mediated by plasma factors.

### Fatal Outcome Is Accompanied by Altered Circulating Platelet-Derived Factors

As platelet hypo-reactivity in COVID-19 was influenced by plasma components, we explored disparities in plasma composition between patients with different outcome. Using plasma obtained at study day 0 we measured an array of soluble mediators that are known to influence or be released upon platelet activation.

**Figure 4:**
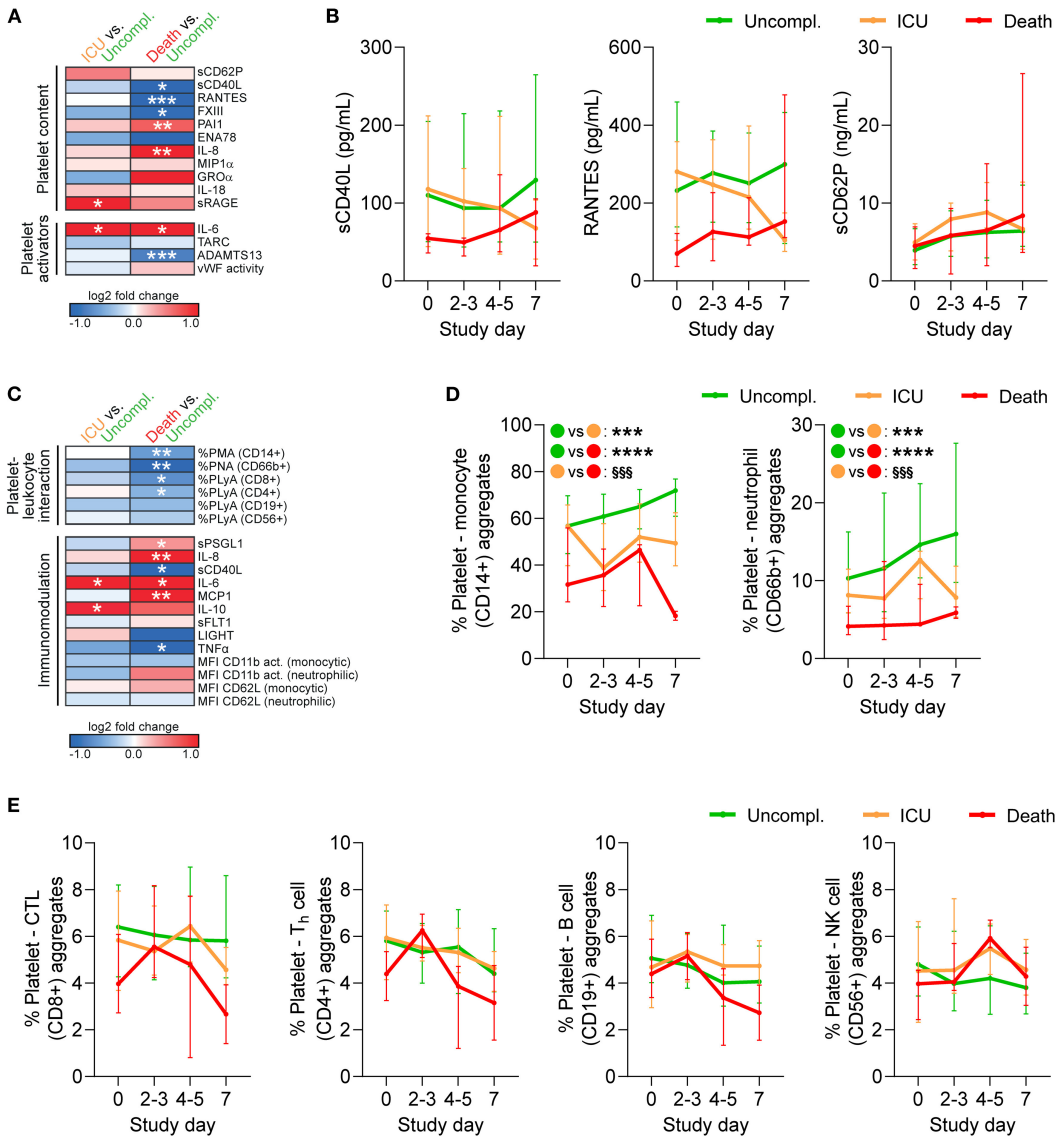
Reduced platelet-derived plasma factors and platelet-leukocyte interaction in COVID-19 patients with fatal outcome. Whole blood (*n* = 97 patients) and plasma (*n* = 106 patients) was analyzed by flow cytometry, multiplex analysis and ELISA **(A,B)** for platelet-contained and platelet-activating mediators and **(C-E)** for platelet-leukocyte aggregates (PLA) and markers of platelet-mediated immunomodulation. **(A,C)** Heatmap visualization of expression profiles at study day 0 in patients requiring ICU treatment or with fatal outcome relative to patients with uncomplicated disease. Data are represented as log2-fold change relative to uncomplicated disease. **(B)** Plasma levels of soluble CD40L (sCD40L), regulated upon activation, normal T cell expressed and secreted (RANTES/CCL5) and sCD62P were monitored over 1 week after study entry. **(D,E)** Percentages of PLA in whole blood were monitored over 1 week after study entry. **(D)** PLA formation with CD14^+^ monocytes and CD66b^+^ neutrophils. Asterisks (^*^) indicate significant differences to uncomplicated, section signs (^§^) indicate significant differences between ICU and death. **(E)** PLA formation with CD56^+^ natural killer (NK) cells, CD19^+^ B cells, CD8^+^ cytotoxic T-lymphocytes (CTL) or CD4^+^ T-helper (T_h_) cells. *n* = 106 (plasma content) or 97 (cell analysis) patients. MFI, mean fluorescence intensity; PMA, platelet-monocyte aggregates; PNA, platelet-neutrophil aggregates; PLyA, platelet-lymphocyte aggregates. ^*^p < 0.05, ^**^p < 0.01, ^***^p < 0.001, ^****^p < 0.0001; ^§§§^*p* < 0.001.

Overall, patients with complicated disease (ICU, death) showed raised levels of pro-inflammatory cytokines and chemokines, which are mainly produced by activated immune cells, but may also derive from platelets. Interleukin 6 (IL-6) was significantly elevated in complicated relative to uncomplicated cases, whereas a disintegrin and metalloprotease with thrombospondin type 1 motifs member 13 (ADAMTS13) was significantly reduced in fatal disease (Figure 4A), both able to augment platelet activation. However, the platelet-derived factors sCD40L, regulated upon activation, normal T cell expressed and secreted (RANTES/CCL5), and coagulation factor XIII (FXIII) were significantly reduced in fatally-ill patients (Figure 4A). Notably, sCD40L and RANTES remained low in fatal disease with little convergence toward uncomplicated cases over time. In contrast, sCD62P which is also released by endothelial cells (24) was not associated with disease outcome (Figure 4B). Therefore, while death was associated with enhanced basal platelet activation and increased soluble platelet activators at study entry, this was not reflected in platelet-released products.

### Reduced Platelet-Leukocyte Aggregates in Patients With Fatal Disease

Finally, we quantified circulating platelet hetero-aggregates with different leukocyte subtypes as well as a panel of functional leukocyte markers that are regulated by platelets. We found that upon study entry, neither monocyte nor neutrophil CD11b activation or CD62L shedding significantly differed between outcomes. However, fatally-ill patients presented with enhanced circulating levels of pro-inflammatory cyto- and chemokines relative to uncomplicated cases, such as elevated IL-8 and monocyte chemoattractant protein 1 (MCP1/CCL2), as well as increased soluble P-selectin glycoprotein ligand 1 (sPSGL1) (Figures 4A,C).

Further, patients with fatal outcome displayed significantly reduced circulating platelet-leukocyte aggregates (PLA), especially of innate leukocytes or T-cells, while ICU patients only showed slight and non-significant reductions relative to uncomplicated disease (Figure 4C). During disease progression both platelet-monocyte aggregates and platelet-neutrophil aggregates remained low in fatal cases, but slightly increased in uncomplicated cases (Figure 4D). Platelets preferentially bind to leukocytes of the myeloid lineage (25). Accordingly, circulating platelet-lymphocyte aggregates involving CD8+ or CD4+ T cells, CD19+ B cells and CD56+ natural killer cells showed only slight variation between outcomes upon study entry (Figure 4C) and comparable kinetics (Figure 4E).

## Discussion

In this prospective study we monitored *in vivo* platelet activation and *in vitro* reactivity in hospitalized COVID-19 patients to investigate dynamic changes with potential influence on patient outcome. We found that adverse outcome was associated with increased platelet activation and diminished platelet responsiveness which aggravated with disease progression. In particular, the ability to activate GPIIb/IIIa was strongly affected in patients who died while ICU patients largely resembled uncomplicated cases regarding platelet dysfunction. In line, circulating platelet-leukocyte aggregates were also diminished in platelets in fatally-ill patients.

Our data on elevated basal platelet activation in COVID-19 non-survivors corroborate and expand on previous findings that link raised expression of platelet degranulation markers, surface receptors and GPIIb/IIIa activation with increased COVID-19 severity (12, 15, 18). However, *ex vivo* agonist-induced platelet aggregation appears to be unaffected by disease severity (17, 18, 21), arguing against enhanced pro-thrombotic platelet capacity with adverse outcome. Hyper-activated platelets are often considered pro-thrombotic and more susceptible to further stimulation. However, in COVID-19 we detected profound and exacerbating platelet hypo-reactivity in fatal COVID-19, particularly regarding integrin activation. Platelet degranulation and integrin activation are regulated by different arms of a highly complex and interwoven intracellular signaling network and while platelet dysfunction often affects both of these cellular functions, the differential regulation observed in our study could point to an involvement of specific inside-out signaling mediators that are primarily attributed to integrin activation. Additionally, impaired GPIIb/IIIa activation is very likely to also reduce outside-in signaling. However, this hypothesis requires further in-depth analysis. Although seemingly counterintuitive, our data underline previously reports showing impaired agonist responses in acutely-ill COVID-19 patients relative to convalescent or healthy controls despite elevated basal activation (17, 19, 20, 26). These findings suggest that platelet hypo-responsiveness is a common feature in COVID-19 which aggravates with disease progression and is associated with increased severity and unfavorable outcome.

Platelet hypo-reactivity in fatal disease is often regarded as a sign of platelet exhaustion, e.g., as described in virologically controlled HIV infection (27). Similarly, activated platelets may be sequestered to the endothelium, rendering the remaining circulating platelets overall hypo-reactive.

The pro-inflammatory milieu in COVID-19 leads to tissue injury, endotheliopathy and extensive immune responses that could provide continuous stimulation for platelets. Indeed, plasma of severely-ill COVID-19 patients stimulates platelet degranulation (15), an effect that may involve engagement of platelet IgG receptor FcγRIIA and/or complement receptor C5aR (26). Further, platelet-activating immune complexes, potentially containing antibodies against SARS-CoV-2, were identified in critically-ill COVID-19 patients (28). Of note, serum IgG levels were found to be lower in severe COVID-19 than in mild/moderate cases or healthy individuals, while complement factor C5a is increased in COVID-19 irrespective of disease severity (29). Thus, while immune complexes and complement induction are likely to bolster platelet hyper-activation in COVID-19, their role for outcome-specific differences in platelet function remains unclear. SARS-CoV-2 also directly induces platelet activation and aggregation through the interaction of angiotensin-converting enzyme 2 (ACE2) receptor and virus spike protein (12), though ACE2 may be circumvented by internalization of microparticle-bound virions (30). Accordingly, basal platelet activation is higher in patients with detectable viral load in blood (12).

However, the fact that neither platelet counts nor plasma levels of platelet-derived sCD40L and RANTES were associated with disease outcome in our study argues against platelet exhaustion or sequestration underlying hypo-reactivity, although they cannot be ruled out. In contrast, we could elucidate that platelet hypo-reactivity in aggravated COVID-19 is at least partially mediated by plasma components, though we were unable to identify the underlying mechanistic mediators.

COVID-19 is associated with dysregulation of platelet receptors for adhesion and agonist responses such as GPVI, GPIb and GPIIb/IIIa (20). COVID-19 patients show elevated plasma levels of soluble GPVI (sGPVI), the ectodomain of the collagen receptor GPVI, which is shed upon platelet activation (19). This was also confirmed *in vitro* studies (31) and sGPVI could potentially interfere with platelet responses to GPVI stimulation. However, sGPVI levels do not increase with disease severity (31) and baseline surface levels of GPVI are not affected in COVID-19 (20, 32). Thus, the contribution of GPVI dysregulation to platelet hypo-responsiveness in fatal outcome remains unclear.

Similar to GPVI, the glycocalicin ectodomain of the vWF receptor GPIbα is also cleaved upon activation and could affect platelet responsiveness. However, reports on altered surface GPIbα expression in COVID-19 are inconsistent (20, 32) and currently no data on glycocalicin are available. Of note, platelet PAR1 expression is not regulated in COVID-19 (20), indicating that platelet hypo-responsiveness in aggravating COVID-19 is not caused by reduced agonist receptor expression.

While initially elevated platelet expression of integrins GPIIb (CD41) and GPIIIa (CD61) was reported in COVID-19 (20), a larger study found no GPIIb/IIIa alterations in acute or convalescent COVID-19 patients (32). We recently found a reduction of certain GPIIb proteoforms in COVID-19 patients with levels further declining in non-survivors over time (33). This might contribute to diminished GPIIb/IIIa responses in fatal outcome. Of note, since we used flow cytometry to quantify the active conformation of GPIIb/IIIa, diminished GPIIb/IIIa activation levels could theoretically also be influenced by a reversal of the receptor to its inactive conformation following previous activation or by a blockage of antibody-binding by a plasma component.

Of note, fatal outcome was associated with elevated levels of several α-granule-derived proteins including IL-8 and plasminogen activator inhibitor 1 (PAI-1). PAI-1 is elevated in sepsis, acute respiratory disease syndrome as well as COVID-19 (34, 35). High plasma levels of PAI-1 in severely-ill COVID-19 patients are associated with reduced fibrinolytic activity, which may promote thromboembolic complications (36). Therefore, PAI-1 is associated with disease severity (37) and predictive for mortality (38). While platelets represent the largest reservoir for PAI-1 under physiological conditions, their contribution to circulating PAI-1 in inflammatory diseases is controversial (39). Especially during systemic infections and endotheliopathy such as COVID-19 endothelial cells produce and secrete PAI-1 (40). Thus, elevated levels of plasma PAI-1 in fatal disease are more likely to indicate endothelial activation than platelet degranulation.

Platelet dysfunction and disease severity may also be linked *via* immune regulation. Direct and indirect platelet-leukocyte interactions typically enhance immune responses such as leukocyte activation and recruitment, thereby contributing to pathogenesis of vascular and infectious diseases (41, 42). We found that platelet degranulation was only mildly impaired in fatal COVID-19, leading to minor alterations in the release of immunomodulatory sCD40L and RANTES. In contrast, platelet binding to monocytes, neutrophils and T-cells was curtailed, suggesting putative impairment of direct platelet-mediated modulation of leukocyte function. Consequently, platelet hypo-responsiveness and concomitant restricted platelet-leukocyte interplay may impact immune responses involved in viral defense or tissue injury. While this may result in impaired immune defenses, platelet hypo-responsiveness in severe infections may also represent a protective host response to limit leukocyte trafficking.

Platelet dysfunction in COVID-19 is a highly dynamic process and time of sampling is crucial. This may at least in part explain the variable results described in different reports. Studies investigating any one time point are inevitably challenged by considerable inter- and intra-study variability and are inherently only able to show a snapshot of pathogenesis which may not reflect physiologic deregulations critical for eventual outcome. Additionally, conflicting findings may also be influenced by individual decisions to seek medical care, local or national hospitalization policies and available capacities of the healthcare system, which are likely to impinge on patient cohort compositions. Overcoming these challenges in patients, omics analyses of rhesus macaques infected with SARS-CoV-2 revealed a rapid induction of platelet activation after infection which declined after several days (43), substantiating our findings regarding dynamic changes in platelet hyper-activation.

Of note our study has certain limitations; as it reports a single center analysis and only includes 11 deceased patients, the observed effects should be validated in independent and larger cohorts. The current study does not comprise virus variants which may have a different impact on platelet function. Moreover, endothelial dysfunctions which also crucially impact on primary hemostasis and thus contribute to thrombotic and hemostatic complications were not investigated, neither were effects of or on the complement system. We also cannot rule out that platelet-leukocyte aggregates became sequestered and could therefore not be detected. Finally, analyses should be repeated in patient cohorts of other viral infections in order to determine if the observed effects are specific for COVID-19.

Taken together our study reveals a hypo-responsive platelet phenotype in COVID-19 patients with adverse outcome which is at least in part mediated by plasma components, though contributions of platelet exhaustion cannot be ruled out. This hints toward a SARS-CoV-2 mediated mechanism to escape entrapment by activated platelets. Diminished platelet reactivity might explain, why anticoagulation can be beneficial in COVID-19 (44), while no effect of anti-platelet therapy could be observed in a randomized trial (45). In contrast, platelet hypo-reactivity may affect their hemostatic capacity, contributing to an increased risk of hemorrhagic complications in critically-ill patients, which might be further exacerbated by anti-platelet medication. Ultimately, platelet hypo-reactivity in fatal COVID-19 patients may also affect platelet-mediated immune responses crucial for viral defense and/or tissue damage.

## Data Availability Statement

The original contributions presented in the study are included in the article/Supplementary Material, further inquiries can be directed to the corresponding author.

## Ethics Statement

The studies involving human participants were reviewed and approved by Ethics Committee of the Medical University of Vienna (EK1315/2020, EK1548/2020). The patients/participants provided their written informed consent to participate in this study.

## Author Contributions

WS, BJ, and AA: contributed to study conception. WS, AP, DP, AS, LB, AK, and JR: performed experiments. WS, AP, SH, HH, and LB: analyzed data. JS, KK, JO, EP, ST, MP, MT, CS, TS, MK, and AZ: recruited and treated patients and provided data. WS and AA: wrote the manuscript. AA: supervised the study. All authors contributed to the article and approved the submitted version.

## Funding

AK is a Wellcome Funded Sir Henry Fellow (218649/Z/19/Z). JR is a British Heart Foundation Intermediate Fellow (FS/IBSRF/20/25039). This work is part of the ACOVACT study of the Medical University of Vienna and is financially supported by grants of the Austrian National Bank to WS (OENB18450) and of the Austrian Federal Ministry of Education, Science and Research, the Medical-Scientific Fund of the Mayor of Vienna (COVID024), and the Austrian Science Fund (P32064, P34783, SFB-54) to AA.

## Conflict of Interest

The authors declare that the research was conducted in the absence of any commercial or financial relationships that could be construed as a potential conflict of interest.

## Publisher's Note

All claims expressed in this article are solely those of the authors and do not necessarily represent those of their affiliated organizations, or those of the publisher, the editors and the reviewers. Any product that may be evaluated in this article, or claim that may be made by its manufacturer, is not guaranteed or endorsed by the publisher.
