# Supplementary material for: Adverse Outcome in COVID-19 Is Associated With an Aggravating Hypo-Responsive Platelet Phenotype
Source: Front Cardiovasc Med. 2021 Dec 10;8:795624. doi: 10.3389/fcvm.2021.795624 (PMC8702807; doi:10.3389/fcvm.2021.795624)
Supplement: Supplementary file 1 [file Data_Sheet_1.PDF]

Supplementary Figures

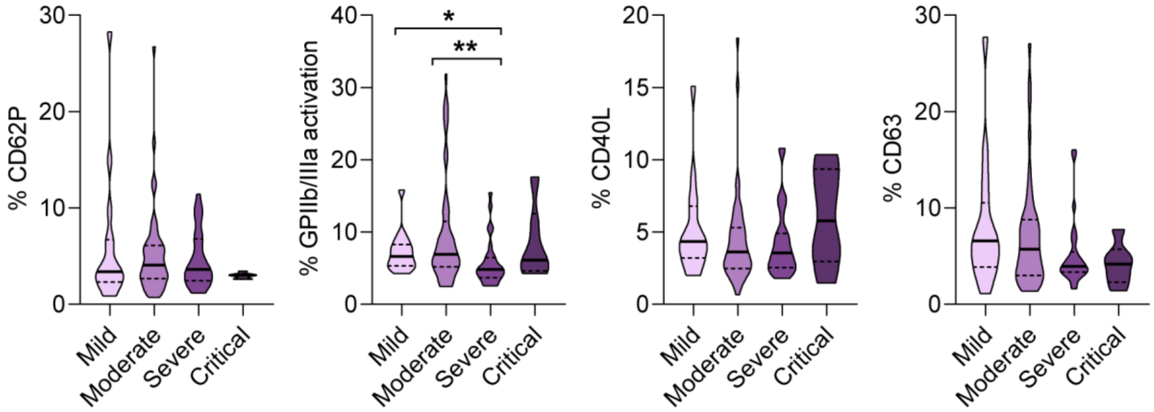

Supplementary Figure 1: Disease severity upon admission is not associated with changes in platelet activation. Patients were stratified upon admission (guidelines issued by the World Health Organization). Basal platelet activation upon study entry was assessed in whole blood via flow cytometry by quantifying surface expression of degranulation markers CD62P, CD40L and CD63 as well as measurement of GPIIb/IIIa activation (PAC1 antibody binding). n=97 patients.*p<0.05, **p<0.01.

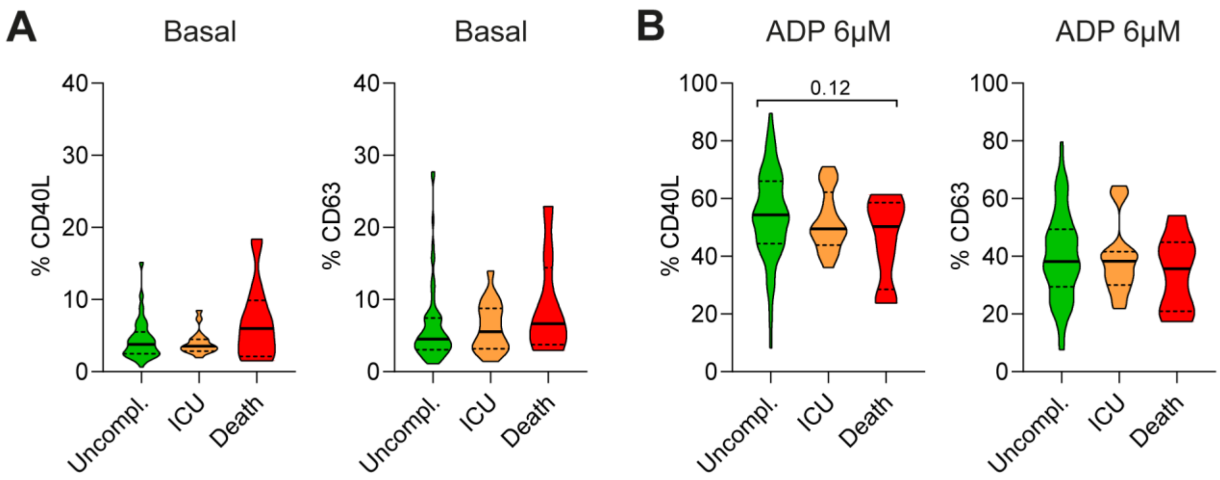

Supplementary Figure 2: Association of COVID-19 disease outcome with altered CD40L and CD63 expression. Platelet degranulation upon study entry at **(A)** basal condition and **(B)** upon stimulation with 6µM ADP (15 min) was assessed in whole blood via flow cytometry by quantifying surface expression of CD40L (left panels) and CD63 (right panels). n=97 patients.

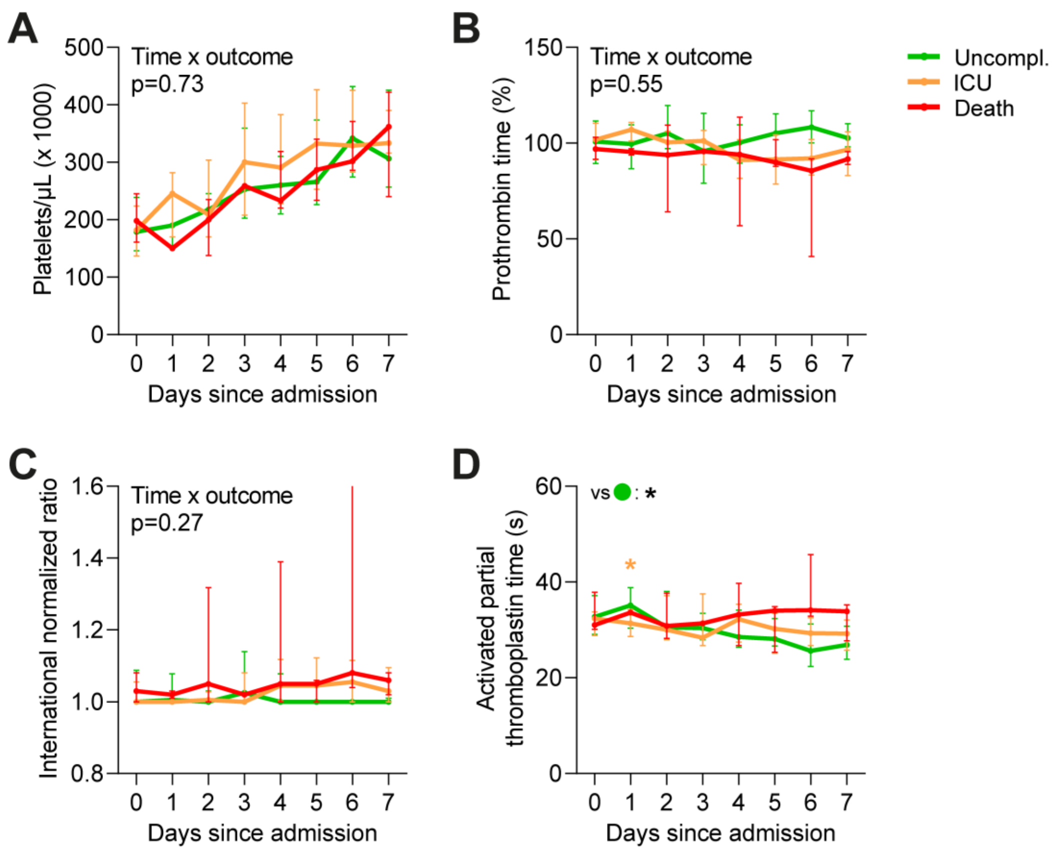

Supplementary Figure 3: Kinetics of platelet count and coagulation parameters in COVID-19 patients with different outcome. Laboratory parameters were analysed in COVID-19 patients over the first 7 days after hospital admission and compared between patients with different outcome. **(A)** Platelet count. **(B)** Prothrombin time. **(C)** International normalized ratio. **(D)** Activated partial thromboplastin time. n=110 patients. Asterisk (*) indicates significant differences to uncomplicated (orange: ICU) at indicated time point. *p<0.05.

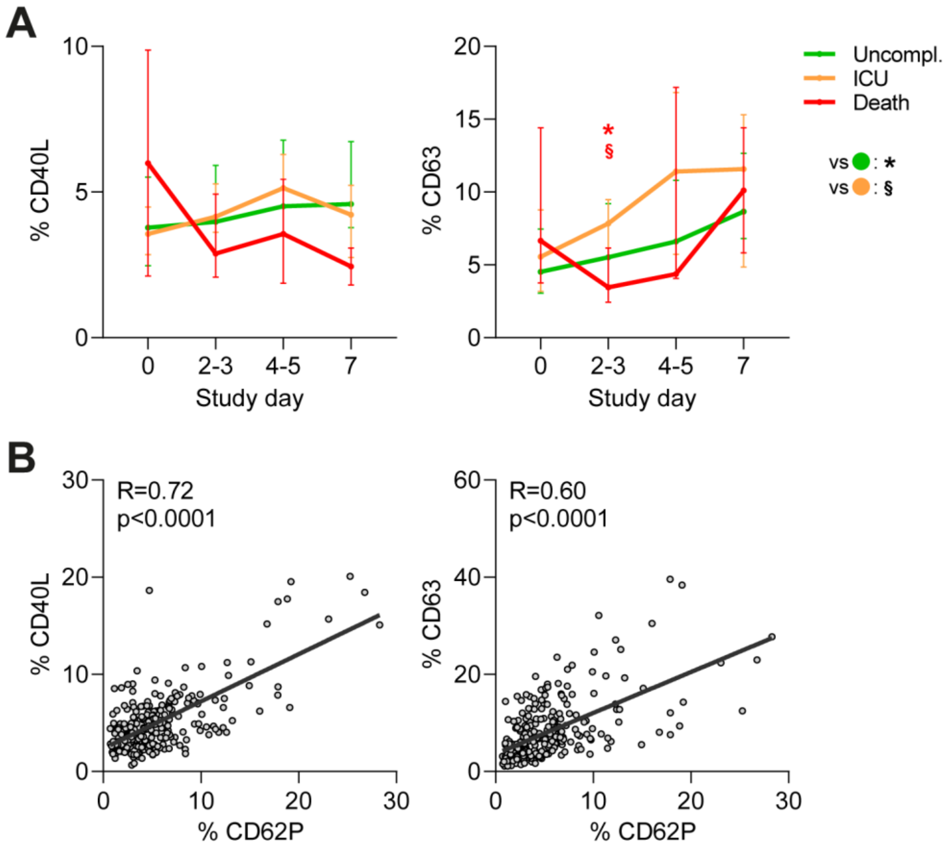

Supplementary Figure 4: Association of COVID-19 disease outcome with kinetics of platelet CD40L and CD63. (A) Basal platelet degranulation over the span of one week after study entry was assessed in whole blood by quantifying surface expression of CD40L (left panel) and CD63 (right panel). **(B)** Correlation of basal CD62P levels with CD40L (left panel) and CD63 (right panel) over all time points. n=97 patients (see Fig. 1A). Asterisks (*) indicate significant differences to uncomplicated (red: death), section signs (§) indicated significant differences between ICU and death. *p<0.05; §p<0.05.

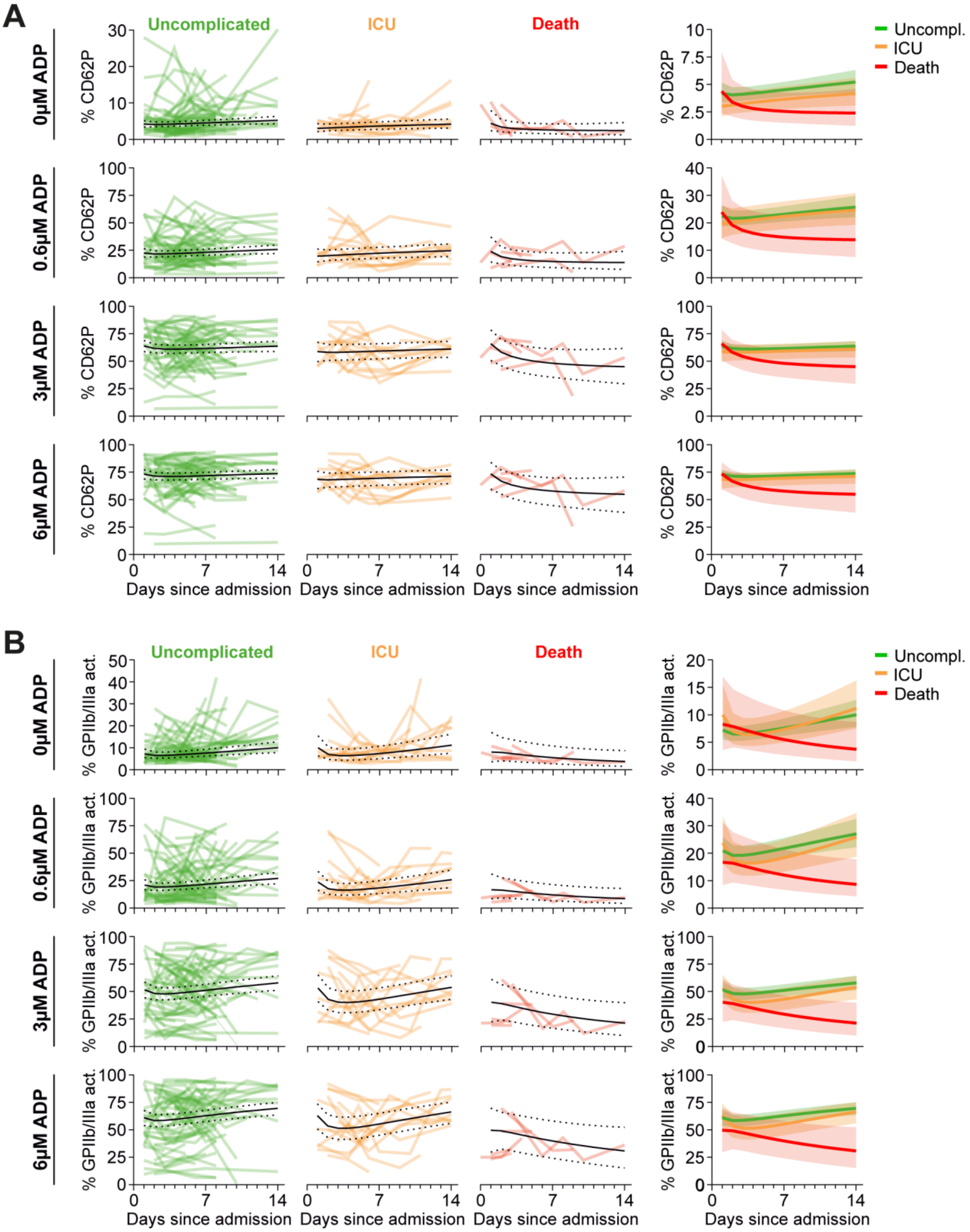

Supplementary Figure 5: Mixed model of ADP-triggered platelet activation over time in patients according to outcome. Platelet activation at basal condition and in response to ADP stimulation (0.6-6µM) was monitored in 97 patients via flow cytometry by quantifying surface expression of CD62P and GPIIb/IIIa activation (PAC1 binding). A mixed model approach was then used to estimate the different kinetics of platelet activation and reactivity between patients with different outcomes and over time according to individual

hospitalization timeline. The model was applied to independently explore **(A)** ADP-triggered CD62P expression and **(B)** ADP-triggered GPIIb/IIIa activation as measured by PAC1 binding. Spaghetti Plots show courses of individual patients (colored lines) with central tendencies of patient groups (black line) and their 95% confidence intervals (dotted lines). Summary plots show an overlay of central tendencies (colored lines) and confidence intervals (shaded areas). Green: Uncomplicated, orange: ICU, red: death. Outcome-specific time courses did not differ between agonist concentrations (CD62P: p=0.28; GPIIb/IIIa: p=0.99).

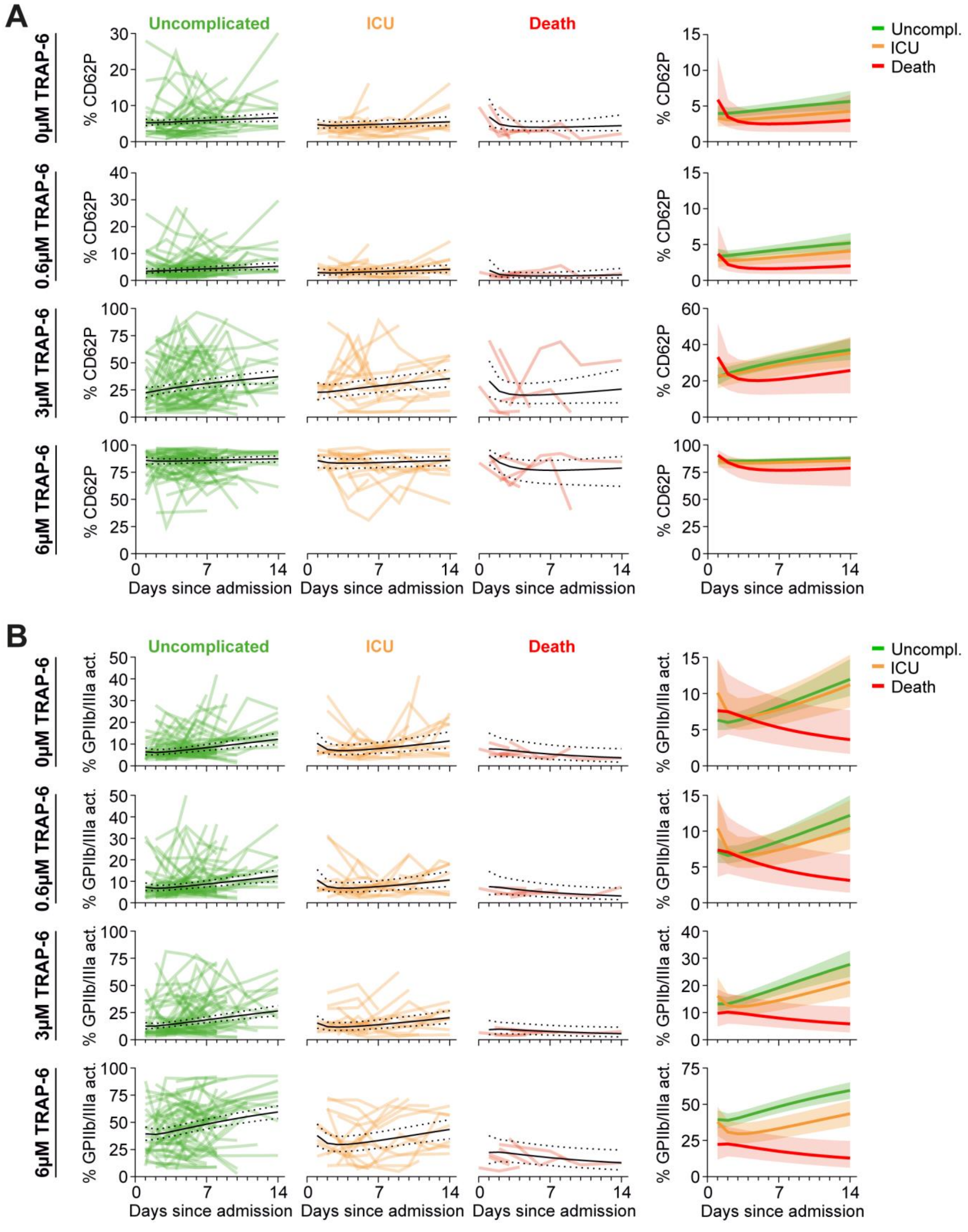

Supplementary Figure 6: Mixed model of TRAP-6-triggered platelet activation over time in patients according to outcome. Platelet activation at basal condition and in response to TRAP-6 stimulation (0.6-6µM) was monitored in 97 patients via flow cytometry by quantifying surface expression of CD62P and GPIIb/IIIa activation (PAC1 binding). A mixed model approach was then used to estimate the different kinetics of platelet activation and reactivity between patients with different outcomes and over time according to individual

hospitalization timeline. The model was applied to independently explore **(A)** TRAP-6-triggered CD62P expression and **(B)** TRAP-6-triggered GPIIb/IIIa activation as measured by PAC1 binding. Spaghetti Plots show courses of individual patients (colored lines) with central tendencies of patient groups (black line) and their 95% confidence intervals (dotted lines). Summary plots show an overlay of central tendencies (colored lines) and confidence intervals (shaded areas). Green: Uncomplicated, orange: ICU, red: death. Outcome-specific time courses did not differ between agonist concentrations (CD62P: p=0.50; GPIIb/IIIa: p=0.81).

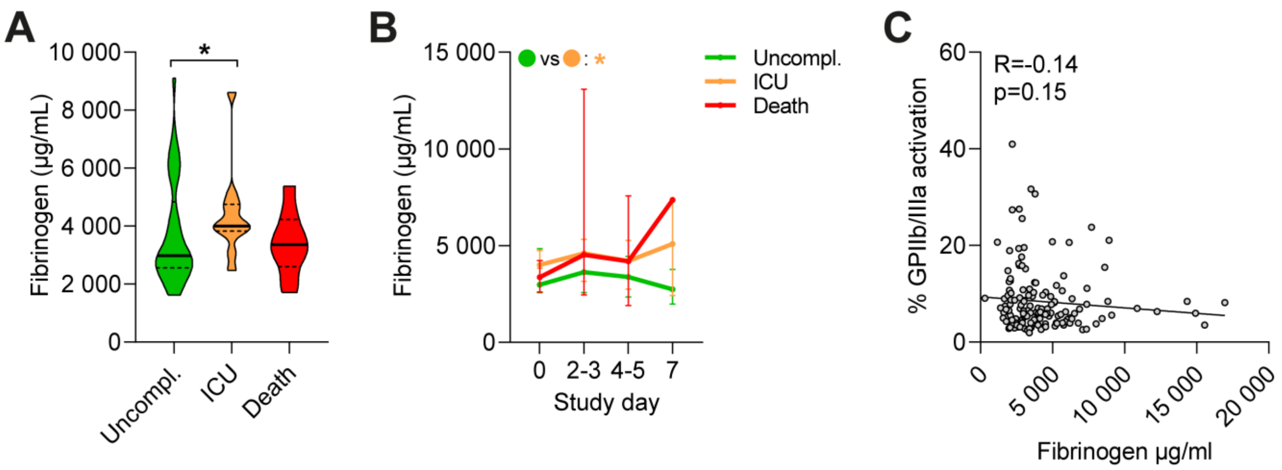

Supplementary Figure 7: Plasma fibrinogen levels do not associate with worsening disease outcome and do not correlate with platelet GPIIb/IIIa activation. Plasma levels of fibrinogen were measured in a subset of patients using multiplex analysis. **(A)** Plasma fibrinogen levels were analyzed at day 0. **(B)** Plasma fibrinogen levels were monitored over one week after study entry. **(C)** Correlation of plasma fibrinogen with platelet GPIIb/IIIa activation was examined over all time points. n=54 patients (34x uncomplicated, 11x ICU, 9x Death). Asterisk (*) indicates significant differences to uncomplicated (orange: ICU). *$p<0.05$.

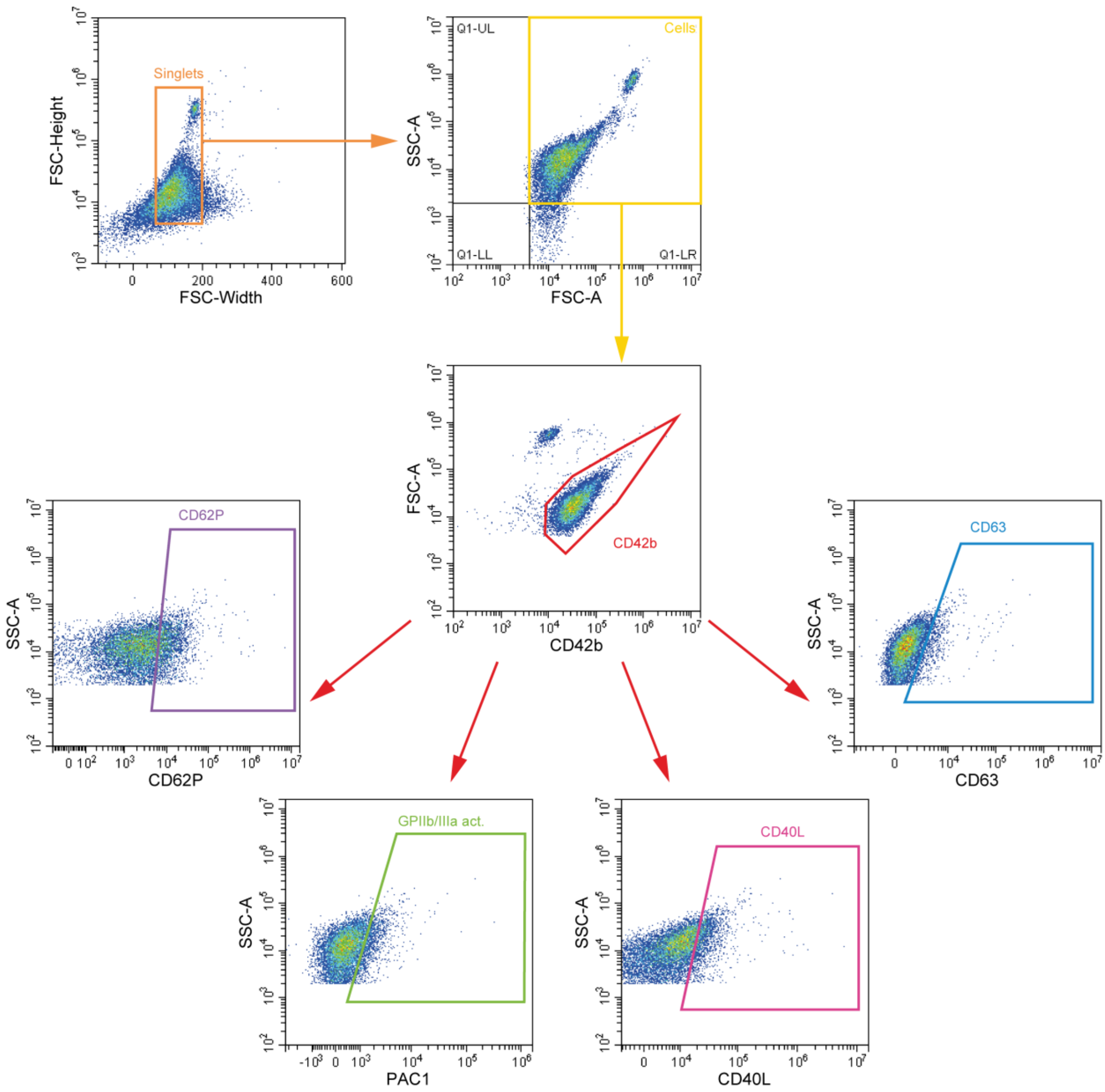

Supplementary Figure 8: Gating strategy to determine platelet activation. Singlet cells with characteristic FSC-A/SSC-A side scatter were gated for CD42b-positivity to identify platelets. Platelets were examined for changes in CD62P, activated GPIIb/IIIa (PAC1-binding), CD40L and CD63.

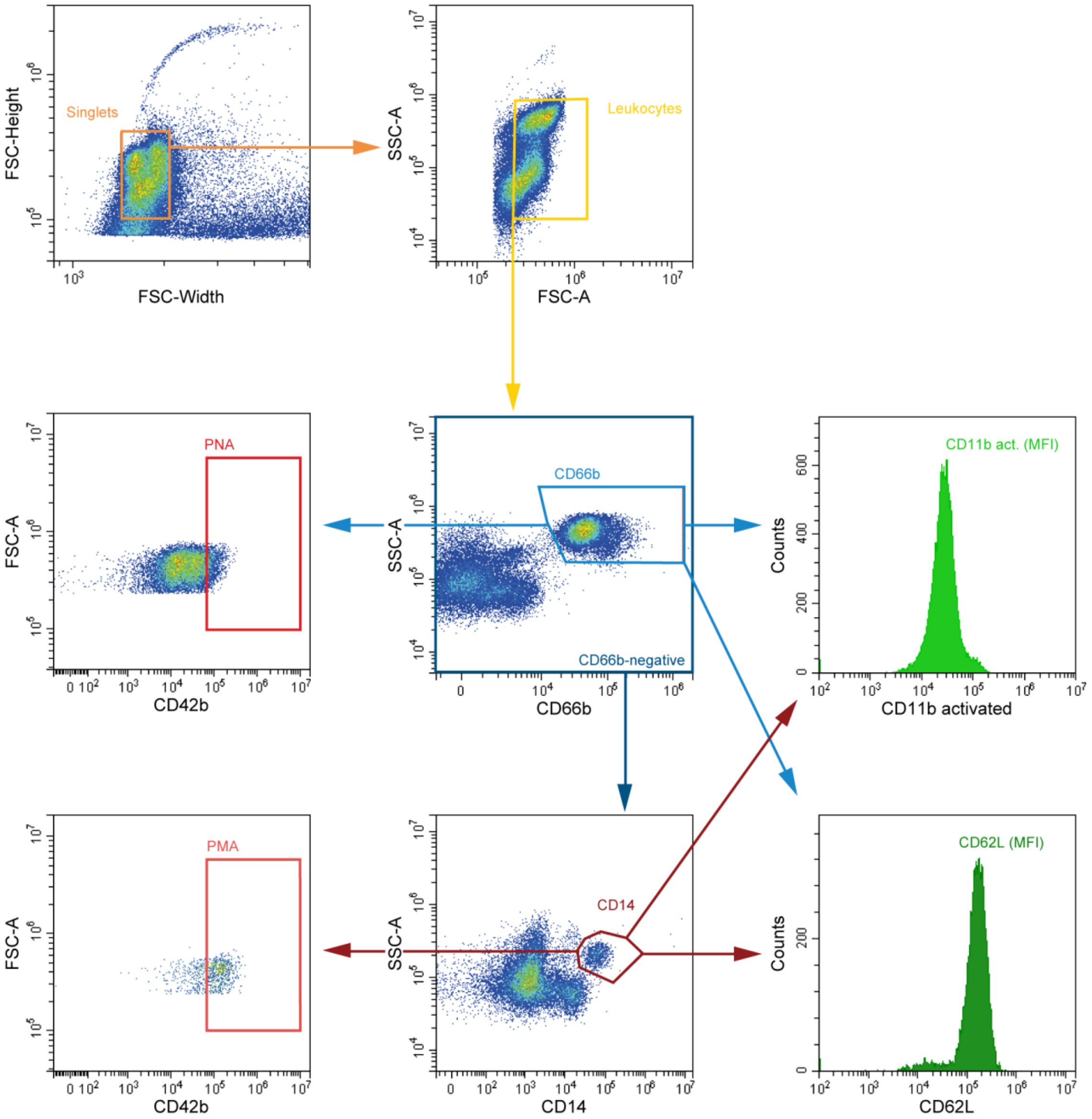

Supplementary Figure 9: Gating strategy to evaluate activation and platelet-binding to neutrophils and monocytes. Singlet cells with characteristic FSC-A/SSC-A side scatter were loosely defined as "leukocytes" and gated for CD66b-positivity to identify neutrophils. CD66b-negative leukocytes were gated for CD14-positivity to identify monocytes. Neutrophils and monocytes were evaluated for CD42b-positivity to identify platelet-neutrophil aggregates (PNA) and platelet-monocyte aggregates (PMA) and for expression of activated CD11b and CD62L.

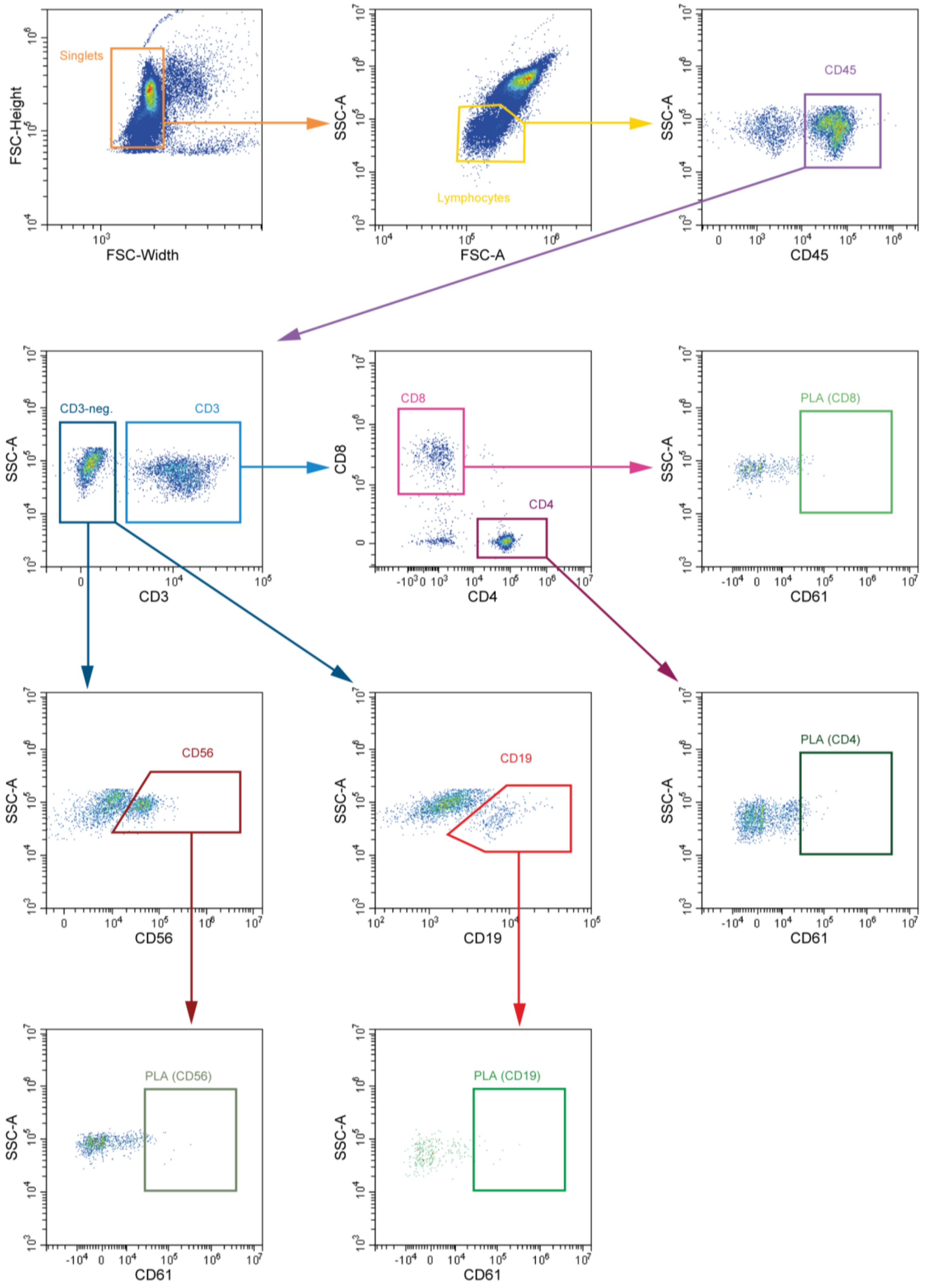

Supplementary Figure 10: Gating strategy to evaluate platelet-binding to lymphocytes and natural killer cells. Singlet cells with characteristic FSC-A/SSC-A side scatter were loosely defined as “lymphocytes” and gated for CD45-positivity to identify small leukocytes. CD45+ cells were gated for CD3 to identify T-cells which were further classified into CD4+ T-helper cells and CD8+ cytotoxic T-cells. CD3- events were gated for CD19 to identify B-cells and for CD56 to identify natural killer (NK) cells. Lymphocyte subsets and NK cells were evaluated for CD61-positivity to identify platelet-leukocyte aggregates (PLA) of respective leukocyte subpopulations.
