# Supplementary material for: Adverse Outcome in COVID-19 Is Associated With an Aggravating Hypo-Responsive Platelet Phenotype
Source: Front Cardiovasc Med. 2021 Dec 10;8:795624. doi: 10.3389/fcvm.2021.795624 (PMC8702807; doi:10.3389/fcvm.2021.795624)
Supplement: Supplementary file 2 [file Data_Sheet_2.PDF]

1 Supplementary Methods

1.1 Patient medication

Standard treatment of hospitalized COVID-19 patients included prophylactic anticoagulation. Patients in the general ward received low-molecular weight heparin (nadroparin) at normal dosage (<70 kg/glomerular filtration rate (GFR) ≥30: 0.4 mL; <70 kg/GFR < 30: 0.3 mL; >70 kg/GFR ≥30: 0.6 mL; >70 kg/GFR <30: 0.4 mL), patients in the ICU received high-dose prophylaxis with nadroparin (<70 kg/GFR>30: 30 mg 2x daily; >70 kg/GFR>30: 40 mg 2x daily; >100kg/GFR>30: 60 mg 2x daily) or enoxaparin (GFR<0: 20 mg 2x daily). Newly diagnosed thromboembolic events, suspected pulmonary emboli were treated by therapeutic anticoagulation, which was also taken into consideration at D-dimer >3 mg/dL. Patients treated with anti-platelet therapy received either 100 mg aspirin (daily), 75 mg clopidogrel (2x daily), 90 mg ticagrelor (2x daily) or a combination thereof. Long-term therapeutic medication including anticoagulants and anti-platelet medication was continued.

1.2 Gating strategies

Platelet panel: Platelets were identified as singlet cells with characteristic forward (FSC) and side scatter (SSC) and CD42b positivity. Expression of CD62P, CD63, and CD40L and GPIIb/IIIa activation (PAC-1 binding) was determined as % of platelets (Supplementary Figure 8).

Innate panel: Singlet cells with characteristic FSC and SSC properties were loosely identified as leukocytes. Neutrophils were identified as CD66b-positive leukocytes. Monocytes were identified as CD66b-negative leukocytes that express CD14. Formation of platelet-leukocyte aggregates was quantified as % of neutrophils or monocytes, surface expression of activated CD11b and CD62L was quantified as mean fluorescence intensity (MFI) of respective subpopulation (Supplementary Figure 9).

Adaptive panel: Singlet cells with characteristic FSC and SSC properties and CD45 positivity were loosely identified as lymphocytes. T-cells were identified as CD3-positive lymphocytes and sub-classified as CD4-positive T-helper cells and CD8-positive cytotoxic T-cells. CD3-negative lymphocytes were analyzed for expression of CD19 and CD56 to identify B-cells and natural killer cells, respectively. All identified subsets were analyzed for CD61 positivity to determine platelet-leukocyte aggregate formation, quantified as % of respective subpopulation (Supplementary Figure 10).

1.3 Modelling of platelet activation/reactivity kinetics

To explore whether platelet activation and reactivity might develop differently over time between the three clinical outcomes uncomplicated, ICU, and death, a mixed model approach was applied four times: i) for ADP-triggered CD62P expression, ii) TRAP-6-triggered CD62P expression, iii) for ADP-triggered GPIIb/IIIa activation (i.e. PAC1-binding), iv) TRAP-6-triggered GPIIb/IIIa activation.

The dependent variables CD62P and PAC1 were quantified by flow cytometry, which implied a gating strategy leading to a percentage of positive cells in each sample. The resulting boundaries of the distribution at 0% and 100% made a logit data transformation necessary to stabilize the residual distribution of the models.

The variables used to specify fixed effects were i) the clinical outcome as factor with three levels, ii) the concentration of the platelet agonists as factor with the four levels 0µM, 0.6µM, 3µM and 6µM, iii) a log-transformed continuous time variable with the original unit days, and iv) the patient's age. v) To allow a curved time course of platelet activation, a log(time)*log(time) variable was also induced as predictor. The dependency of values taken from each patient were accounted for by including a random factor with patients as levels, introducing a random intercept.

The modelling approach started with a full factorial model, from which interactions were removed if $p>0.05$, starting with the highest-order interactions. Interactions were not removed if they were part of a significant higher order interaction. This strategy was followed until only significant interactions were left, which were then interpreted. E.g., if significant, the interaction 'clinical outcome*agonist concentration*log(time)' would indicate that the time course of platelet activation was different between outcomes, which in turn was dependent on the platelet agonist concentration.

In the final models, contrasts were used to estimate mean group differences (on the logit transformed data) at specific time points at specific agonist concentrations as well as group means with 95% confidence intervals. The least square estimated logit means were back-transformed and plotted on top of the raw data to allow inspection how well the model suits the data.

Of note, the statistical modeling implied several decisions, e.g. regarding the type of model, including the agonist concentration as levels of a factor or as continuous covariate, random effects, and data transformations to name but a few. In addition, the complex data structure results in several p-values, also slicing the model at certain time points results in a theoretically endless number of p-values. Therefore, the presented results should rather be understood as exploratory analysis of patterns in the data than as rigorous hypothesis testing. In addition, an assumption is a missing at random (MAR) situation, which is plausible as missingness of data can be assumed to be mostly a result of the outcome and age. Furthermore, interpretation of results needs to consider that no adjustment for multiple testing was applied.

Statistical analyses were performed with IBM SPSS 27 using the restricted maximum likelihood method, diagrams were generated in GraphPad Prism 8 and SPSS. Only two-sided p-values were used, p-values ≤ 0.05 were considered statistically significant.

1.4 Heatmap analysis

Log2-fold changes were calculated for each parameter between patients requiring ICU treatment or with fatal outcome relative to patients with uncomplicated disease. Differences were tested using two-sided Wilcoxon rank sum test using R 4.0.1 (The R Foundation for Statistical Computing, Vienna, Austria) and visualized as a heatmap using Genesis (version 1.8.1). Where indicated, p-values were adjusted for multiple testing based on the false discovery rate according to the Benjamini-Hochberg method. Differences with $p<0.05$ were considered as statistically significant.
