# Supplementary material for: Adverse Outcome in COVID-19 Is Associated With an Aggravating Hypo-Responsive Platelet Phenotype
Source: Front Cardiovasc Med. 2021 Dec 10;8:795624. doi: 10.3389/fcvm.2021.795624 (PMC8702807; doi:10.3389/fcvm.2021.795624)
Supplement: Supplementary file 3 [file Data_Sheet_3.PDF]

Supplementary Tables

Supplementary Table 1: Staining panels for flow cytometric analysis of patient whole blood

	Antibody	Fluorophore	Clone	final concentration	Company
Platelet panel	α-CD42b	PerCP	HIP1	1:75	BioLegend
	α-CD62P	Brilliant Violet 605	AK4	1:100	BioLegend
	α-CD63	Pacific Blue	H5C6	1:100	BioLegend
	α-CD40L	PE	24-31	1:50	BioLegend
	PAC-1 (α-GPIIb/IIIa activated)	FITC	PAC-1	1:40	BD Biosciences
Innate panel	α-CD42b	PerCP	HIP1	1:75	BioLegend
	α-CD66b	Pacific Blue	G10F5	1:75	BioLegend
	α-CD14	APC	MΦP9	1:60	BD Biosciences
	α-CD11b activated	FITC	CBRM1/5	1:60	BioLegend
	α-CD62L	Brilliant Violet 650	DREG-56	1:60	BioLegend
Lymphocyte panel	α-CD61	FITC	VI-PL2	1:60	BioLegend
	α-CD45	Brilliant Violet 605	HI30	1:150	BioLegend
	α-CD3	Pacific Blue	UCHT1	1:50	BioLegend
	α-CD4	APC-Cy7	OKT4	1:200	BioLegend
	α-CD8	PE	SK1	1:200	BioLegend
	α-CD19	PerCP	HIB19	1:100	BioLegend
	α-CD56	Alexa Fluor 647	MEM-188	1:50	BioLegend
